# Retraction Note: miR-3928v is induced by HBx via NF-κB/ EGR1 and contributes to hepatocellular carcinoma malignancy by down-regulating VDAC3

**DOI:** 10.1186/s13046-022-02580-2

**Published:** 2022-12-28

**Authors:** Qiaoge Zhang, Ge Song, Lili Yao, Yankun Liu, Min Liu, Shengping Li, Hua Tang

**Affiliations:** 1 Tianjin Life Science Research Center and Department of Pathogen Biology, Collaborative Innovation Center of Tianjin for Medical Epigenetics, School of Basic Medical Sciences, Tianjin Medical University, No. 22 Qi-Xiang-Tai Road, Tianjin, 300070 China; 2 The Cancer Institute, Tangshan People’s Hospital, Tangshan, 063001 China; 3 Department of Hepatobiliary Oncology, State Key Laboratory of Oncology in Southern China, Cancer Center, Sun Yat-sen University, Guangzhou, 510060 China

**Retraction Note: J Exp Clin Cancer Res 37, 14 (2018)**

**https://doi.org/10.1186/s13046-018-0681-y**

The Editor in Chief has retracted this article. After publication, concerns were raised about Figs. 4d, 5e, and 6h. These were addressed in a Correction [1]. Later, a new concern arose around the similarity between two GADPH blots in Fig. 2f. The authors provided raw images, which raised further concerns regarding MMP2 blots. Additionally, concerns have been raised regarding the ethics approval documentation provided by the authors. The Editor in Chief, therefore, has lost confidence in the findings of the paper. All authors agree to this retraction.
